# The prognostic impact of overall treatment time on disease outcome in uterine cervical cancer patients treated primarily with concomitant chemoradiotherapy: a nationwide Taiwanese cohort study

**DOI:** 10.18632/oncotarget.19617

**Published:** 2017-07-27

**Authors:** Shih-Min Lin, Hsiu-Ying Ku, Ting-Chang Chang, Tsang-Wu Liu, Ji-Hong Hong

**Affiliations:** ^1^ Department of Radiation Oncology, Chang Gung Memorial Hospital, Taoyuan, Taiwan; ^2^ National Health Research Institute, Miaoli, Taiwan; ^3^ Department of Obstetrics and Gynecology, Chang Gung Memorial Hospital, Taoyuan, Taiwan

**Keywords:** cervical cancer, concurrent chemoradiation, irradiation prolongation, overall treatment time, radiotherapy

## Abstract

The importance of the overall treatment time (OTT) has a paradoxical status in the current era of concomitant chemoradiotherapy. The main objective of this nationwide study was to evaluate the correlation between overall treatment duration and clinical outcome in cervical cancer patients treated primarily with curative concurrent chemoradiotherapy (CCRT). In this population-based cohort study, 2,594 patients diagnosed with International Federation of Gynecology and Obstetrics (FIGO) stage I-IVA uterine cervical cancer were studied. Univariate and multivariate analyses of prognostic factors were analyzed using Cox’s proportional hazards models. The median irradiation duration was 59 days. Significant prognostic factors related to poor cancer-specific survival (CSS) and overall survival (OS) included old age, non-squamous cell cancer type, high-grade histology, increased tumor size, advanced FIGO stage, and prolonged OTT. After multivariate analysis, prolonged treatment time remained as a significant factor for poor CSS (hazard ratio, HR = 1.33; p < 0.001) and OS (HR = 1.15; p = 0.05). Further subgroup analysis showed that the 5-year OS rates after a treatment time of ≤ 56 days compared with > 56 days in patients with FIGO stages I-IIB and III-IVA were 70% and 65% (p = 0.002) compared with 43% and 42% (p = 0.67), respectively. Inconclusion, completion of CCRT within 8 weeks is recommended, particularly for patients with FIGO stage I-IIB disease.

## INTRODUCTION

Before the era of concurrent chemoradiotherapy (CCRT), the overall treatment time (OTT) was considered to be a critical parameter for pelvic control and survival in cervical cancer patients treated primarily with radiotherapy (RT) [1–4]. An RT course duration of more than 8 weeks results in decreased cause-specific survival and overall survival (OS). The detrimental effect of treatment time prolongation is more prominent in patients with a larger tumor size and International Federation of Gynecology and Obstetrics (FIGO) stage III disease [3, 4]. By analyzing clinical data and using a linear quadratic model to determine the best fit, the onset of accelerated tumor repopulation was estimated to be approximately 19 days after RT treatment, and this might contribute to poor disease control [5, 6]. It has been suggested that OS decreases by 0.6% per day and pelvic control by 0.7% per day for each additional day of treatment beyond 55 days for all stages of disease [3].

The impact of the total treatment time on local control and survival is complex, and contradictory results have been obtained. CCRT using a cisplatin regimen has been suggested to increase RT sensitivity, thereby increasing the biologically effective dose of RT, and several clinical trials have shown a survival advantage for locally advanced cervical cancer [7–10]. In a study involving patients treated by either RT alone or CCRT, an extended total treatment time of > 62 days adversely impacted treatment efficacy in patients treated with RT alone but had no effect on patients treated by CCRT [11]. In another study, a treatment time of > 56 days was found to be detrimental to pelvic control but with no effect on distant metastasis or disease-specific mortality [12]. Hence, the importance of the OTT has a paradoxical status in the current era of CCRT.

Based on a literature search, there are few data on the effects of the OTT in Asian populations, and most of these studies involved the use of limited subpopulation analyses, such as studies composed of limited FIGO stage patients [2]. Thus, this study was initiated to resolve this issue; the national Taiwan Cancer Registry Database, which collects data on more than 95% of cancer patients in Taiwan and contains essential information and follow-up data, was used for the analysis. The aim of this study was to perform a retrospective analysis of patients with stage I–IVA cervical cancer who were treated definitively with RT or CCRT and to evaluate the influence of the OTT on disease-specific survival and OS. To reduce the number of confounding factors arising from treatment selection bias, we used propensity-weighted models. To our knowledge, this study involves the largest series related to this issue and is the first to evaluate the effect of OTT across all FIGO stages in a national population of women with cervical cancer.

## RESULTS

### Patient characteristics

The nationwide population evaluated in the study consisted of 8,968 patients diagnosed with invasive uterine cervical cancer between January 2007 and December 2013. In this cohort, 3,607 patients (40%) were treated primarily with curative RT. After excluding patients with FIGO IVB, 2,594 patients treated with a curative radiation dose remained, and 71% of those patients (*n* = 1,850) received CCRT. Details of the patient characteristics are presented in Table 1. The status of lymph node involvement is available for patients diagnosed after 2009 (*n* = 79). The mean age at diagnosis was 62 years. Most of the patients had FIGO stage IIB or more advanced disease (*n* = 1,734; 66.8%). The median duration of the RT course was 59 days (interquartile range = 53-68 days). The median waiting times, defined as the duration between time of biopsy for diagnosis and the beginning date of CCRT, of patients OTT ≤ 56 days and OTT > 56 days were 24 and 23 days, respectively (p < 0.001). The percentages of patients whose waiting times were < 6 weeks were 82.3% and 87.3% in the subgroups of OTT ≤ 56 days and OTT > 56 days, respectively (p < 0.001). Although a slightly longer waiting time was noted in the subgroup of patients with OTT ≤ 56 days, better CSS (HR = 1.33, p < 0.001; Table 2) and OS (HR = 1.15, p = 0.05; Table 2) outcome were noted in subgroup of OTT ≤ 56 day.

**Table 1:** Clinical and demographic characteristics of patients who underwent an entire course of RT or CCRT stratified by a 56-day treatment period

Variables	Total	≤ 56 days (*n* = 993)		> 56 days (*n* = 1,601)		*p*-value^a^
		*n*	(%)	*N*	(%)	
**Mean age (± SD) at diagnosis (years)**		63.01 ± 13.92		62.27 ± 14.05		0.02^b^
**Age at diagnosis (years)**						0.06
≤ 30	15	6	(0.6)	9	(0.6)	
31-40	98	39	(3.9)	59	(3.7)	
41-50	450	148	(14.9)	302	(18.9)	
51-60	672	262	(26.4)	410	(25.6)	
61-70	500	184	(18.5)	316	(19.7)	
> 70	859	354	(35.6)	505	(31.5)	
**Smoking**						0.18
Yes	86	42	(6.8)	44	(6.7)	
No	955	369	(83.7)	586	(86.8)	
Unknown	1116	441	(9.5)	675	(6.5)	
**FIGO stage**						< 0.001
IA	12	7	(0.7)	5	(0.3)	
IB	538	292	(29.4)	246	(15.4)	
2A	310	134	(13.5)	176	(11.0)	
2B	998	372	(37.5)	626	(39.1)	
3A	92	21	(2.1)	71	(4.4)	
3B	503	138	(13.9)	365	(22.8)	
4A	141	29	(2.9)	112	(7.0)	
**Pelvic lymph node status**						0.84
N0	57	25	(2.5)	32	(2.0)	
N1	22	8	(0.8)	14	(0.87)	
Nx	1,355	519	(52.3)	836	(52.22)	
Unknown	1,160	441	(44.4)	719	(44.91)	
**Histology**						0.77^c^
Squamous cell carcinoma	2,250	860	(86.6)	1,390	(86.8)	
Adenocarcinoma	266	99	(10.0)	167	(10.4)	
Neuroendocrine tumor	3	1	(0.1)	2	(0.1)	
Others	75	33	(3.3)	42	(2.6)	
**Grade**						0.02
Well-differentiated	42	14	(1.4)	28	(1.7)	
Moderately-differentiated	733	286	(28.8)	447	(27.9)	
Poorly-differentiated	603	212	(21.3)	391	(24.4)	
Undifferentiated	24	16	(1.6)	8	(0.5)	
Unknown	1,192	465	(46.8)	727	(45.4)	
**Tumor size (cm)**		3.57 ± 2.24		3.91 ± 4.12		0.02 ^b^
Median (SE)		3.50 ± 0.07		4.00 ± 0.10		
Range		0.05–14		0.05–96		
**Waiting time (Diagnosis to treatment)**						
**Median [IQR] (days)**	23 [16-34]	24 [17-37]		23 [15-33]		< 0.001 ^b^
≤ 6 weeks	2060	759	82.3	1301	87.3	< 0.001
> 6 weeks	35	163	17.7	189	12.7	
**Brachytherapy**						< 0.001
No	377	108	(10.9)	269	(17)	
Yes	2,217	885	(89.1)	1,332	(83)	
**Total brachytherapy dose to point A**						
Median (cGy) [IQR]	3333 [3000-3600]	3200 [2900-3440]		3400 [3067-3733]		<0.001 ^b^
≤ 30 Gy	565	237 (26.8)		328 (24.6)		0.25
> 30 Gy	1652	648 (73.2)		1004 (75.4)		
**Estimated accumulated median dose to point A (range, cGy) [IQR]**						
External beam alone	7020 [6720-7200]	7000 [6300-7040]		7100 [6840-7200]		<0.001 ^b^
With LDR brachytherapy	7200 [6720-7980]	6900 [6660-7980]		7470 [7065-8265]		0.31 ^b^
With HDR brachytherapy	8640 [8100-9133]	8480 [7840-9027]		8740 [8240-9200]		<0.001 ^b^
**Chemotherapy**						< 0.001
No	744	323	(32.5)	421	(26.3)	
Yes	1,850	670	(67.5)	1,180	(73.7)	

RT: radiotherapy; CCRT: curative concurrent chemoradiotherapy; SE: standard error; SD: standard deviation.

^a^*p-*values for the difference between patients with an entire course of RT or CCRT stratified by a 56-day treatment period using the *t*-test for continuous variables and χ^2^ test for categorical variables.

^b^Data are presented as *p*-values from a Mann–Whitney U-test.

**Table 2:** Univariate and multivariate overall and specific survival analyses according to Cox’s proportional hazards model

Variables	Cancer-specific survival								Overall survival							
	Univariate			*p*-value	Multivariate			*p*-value	Univariate			*p*-value	Multivariate			*p*-value
	HR	95% CI			HR	95% CI			HR	95% CI			HR	95% CI		
		Lower	Upper			Lower	Upper			Lower	Upper			Lower	Upper	
**Age (years)**																
≤ 30	1.00								1.00							
30–60	0.77	0.32	1.85	0.56					0.88	0.36	2.12	0.77				
> 60	0.84	0.35	2.02	0.69					1.38	0.57	3.33	0.47				
**FIGO Stage**																
IA–IIB	1.00				1.00				1.00				1.00			
IIIA–IVA	2.87	2.47	3.33	< 0.001	2.46	2.09	2.89	< 0.001	2.27	1.99	2.58	<0.001	2.09	1.82	2.41	< 0.001
**Median tumor size (cm)**	1.02	1.01	1.03	< 0.001	1.02	1.01	1.03	0.01	1.01	1.00	1.03	0.02	1.01	1.00	1.03	0.02
**Overall treatment time**																
≤ 56 days	1.00				1.00				1.00				1.00			
> 56 days	1.67	1.41	1.97	<0.001	1.33	1.12	1.58	< 0.001	1.35	1.18	1.55	< 0.001	1.15	1.00	1.32	0.05
**Histology**																
Squamous cell carcinoma	1.00				1.00				1.00				1.00			
Non-squamous cell carcinoma	1.74	1.44	2.11	< 0.001	1.92	1.58	2.33	< 0.001	1.49	1.25	1.77	< 0.001	1.64	1.38	1.95	< 0.001
**Grade**																
Well-to-moderately differentiated	1.00				1.00				1.00							
Poor-to-undifferentiated	1.23	1.00	1.51	0.05	1.11	0.96	1.15	0.78	1.10	0.92	1.31	0.30				
Unknown	1.09	0.91	1.31	0.34	1.03	0.94	1.12	0.56	1.10	0.94	1.28	0.22				
**Brachytherapy**																
Yes	1.00				1.00				1.00				1.00			
No	1.35	1.15	1.58	< 0.001	1.93	1.60	2.31	< 0.001	2.72	2.35	3.16	< 0.001	1.84	1.57	2.15	< 0.001
**Chemotherapy**																
Yes	1.00				1.00				1.00				1.00			
No	2.90	2.44	3.44	< 0.001	1.46	1.23	1.72	< 0.001	1.93	1.70	2.20	< 0.001	2.04	1.79	2.34	< 0.001

CI: confidence interval; HR: hazard ratio; FIGO: International Federation of Gynecology and Obstetrics.

### Treatment

For treatment planning, 25.6% (*n* = 701) of EBRT planning was conducted using a two-dimensional (2D) planning technique and the remaining 74.4% (*n* = 1,893) used a three-dimensional (3D) planning technique. There were 356 (34.3%) and 345 (20.3%) women treated with 2D planning techniques in the subgroups with OTT ≤ 56 days and > 56 days, respectively. Regarding LDR and HDR brachytherapy, there were seven and six patients treated with LDR brachytherapy in the subgroups of OTT ≤ 56 days and OTT > 56 days, respectively. Most patients (*n* = 2,204) were treated with high-dose rate intracavitary brachytherapy. The median brachytherapy dose to point A was 32 Gy in the subgroup of OTT ≤ 56 days and 34 Gy in the subgroup of OTT > 56 days (p < 0.001; Table 1). In those patients receiving EBRT followed by LDR brachytherapy, the median estimated accumulated dose to point A was 69.0 Gy in the subgroup of OTT ≤ 56 days and 74.7 Gy in the subgroup of OTT > 56 days (p = 0.31; Table 1). In those patients receiving EBRT followed by HDR brachytherapy, the median estimated accumulated dose to point A was 84.8 Gy in the subgroup of OTT ≤ 56 days and 87.4 Gy in the subgroup of OTT > 56 days (p < 0.001; Table 1). In those patients receiving EBRT alone, the median radiation dose to the high-risk clinical target volume was 70.0 Gy in the subgroup of OTT ≤ 56 days and 71.0 Gy in the subgroup of OTT > 56 days (p < 0.001; Table 1). No patient received neoadjuvant chemotherapy in the present cohort. Only 18 patients started to receive chemotherapy after completing RT, and the remaining 1,832 patients received concurrent chemo-radiation as their primary treatment.

Increased age (p = 0.06), less advanced FIGO stage (p < 0.001), smaller tumor size (p = 0.02), and well or moderately differentiated tumor histology (p = 0.02) were observed more frequently in patients receiving curative RT with a short OTT (OTT ≤ 56 days; Table 1). Moreover, in the subgroup of patients receiving curative RT over a duration of ≤ 56 days, there was more frequent use of brachytherapy (p < 0.001) and less concomitant use of chemotherapy (p < 0.001; Table 1). No significant difference was found in OTT across smoking status (p = 0.18), differing pathologies (p = 0.77), or pelvic nodal status (p = 0.84).

In UVA and MVA, factors associated with poor CSS and OS both included an advanced FIGO stage, a larger tumor size, a prolonged OTT, non-squamous cell tumor type, and treatment without brachytherapy or without concurrent chemotherapy (Table 2).

### Impact of a prolonged OTT on CSS and OS

**Figure 1:**
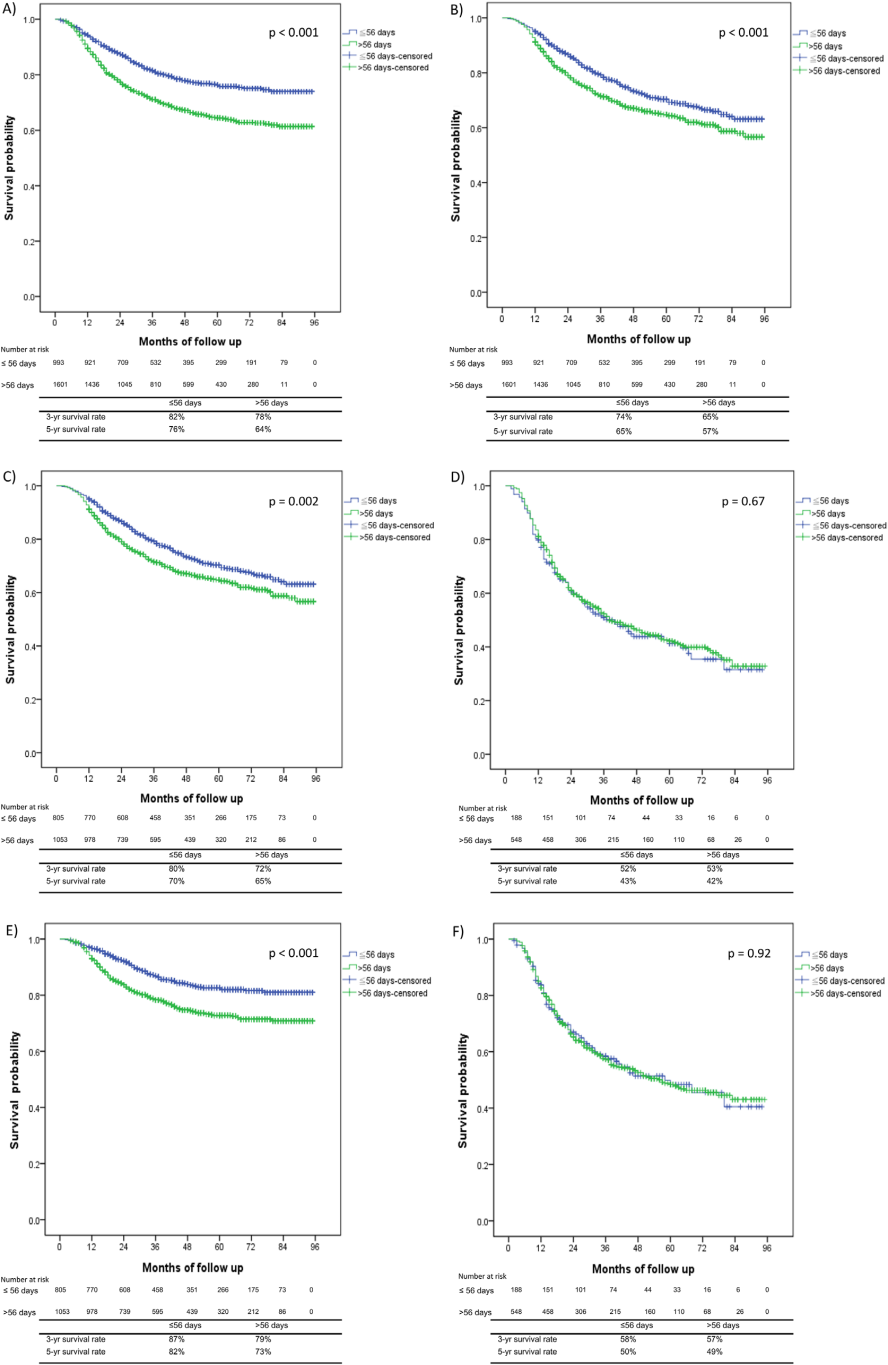
Kaplan–Meier survival curves comparing the patients with an overall treatment time (OTT) ≤ 56 days versus those with an OTT > 56 days **(A)** Cancer-specific survival (CSS) for all patients, **(B)** overall survival (OS) for all patients, **(C)** OS for patients with FIGO stage I-IIB disease, **(D)** OS for patients with FIGO stage III-IVA disease, **(E)** CSS for patients with FIGO stage I-IIB disease, and **(F)** CSS for patients with FIGO stage III-IVA disease.

The mean CSS and OS times in the subgroups with OTT of ≤ 56 days compared with > 56 days were 77.77 and 67.01 months versus 68.61 and 62.34 months, respectively (Table 3). The crude hazard ratios (HRs) for CSS and OS in the subgroup of patients with an OTT of > 56 days were 1.67 (95% confidence interval, CI = 1.41-1.97, p < 0.001) and 1.35 (95% CI = 1.18-1.55, p < 0.001), respectively (Table 3). The 5-year cumulative incidences of CSS for patients with OTT of ≤ 56 days versus OTT > 56 days were 76% versus 64%, respectively (p < 0.001; Figure 1A). The 3-/5-year cumulative incidences of OS for patients with OTT ≤ 56 days compared with OTT > 56 days were 74/65%, compared with 65/57%, respectively (p < 0.001; Figure 1B). OS analysis using the Kaplan–Meier method revealed that every additional treatment day after a 56-day treatment period decreased the 3-year OS rate by 0.8%.

**Table 3:** Impact of the duration of the entire course of RT or CCRT according to Cox’s proportional hazards model

	Mean survival time (months)				95% CI		Cox’s proportional hazards			
	Total (n)	Death (n)	Mean	SE	Lower bound	Upper bound	Crude HR (95% CI)	*p-*value	Adjusted HR (95% CI)^a^	*p-*value^a^
**OS**										
≤ 56 days (Reference)	993	312	67.01	1.18	66.68	71.34	1.00		1.00	
> 56 days	1601	641	62.34	0.96	60.43	64.26	1.35 (1.18–1.55)	0.001	1.08 (0.93–1.24)	0.31
**CSS**										
≤ 56 days (Reference)	993	194	77.77	1.11	75.63	79.91	1.00		1.00	
> 56 days	1601	494	68.61	0.96	66.71	70.5	1.67 (1.41–1.97)	0.001	1.23 (1.04–1.47)	0.02

RT: radiotherapy; CCRT: curative concurrent chemoradiotherapy; HR: hazard ratio; CI: confidence interval; SE: standard error; OS: overall survival; CSS: cancer-specific survival.

^a^Adjusted for age, FIGO stage, histology, requirement for brachytherapy, and requirement for chemotherapy.

We further analyzed the effects of the OTT on CSS and OS after adjusting for confounding factors such as age, FIGO stage, histology, use of brachytherapy, and use of chemotherapy. A poor CSS maintained a significant association with a prolonged OTT > 56 days (HR = 1.23, 95% CI = 1.04-1.47, p = 0.02; Table 3). Focusing on those receiving concurrent chemoradiation and brachytherapy, this subgroup analysis also revealed that prolonged OTT had detrimental effects on OS (HR = 1.29, 95% CI = 1.05-1.58, p = 0.01; Supplementary Table 1) and CSS (HR = 1.29, 95% CI = 1.02-1.62, p = 0.03; Supplementary Table 1).

### Impact of a prolonged OTT on CSS and OS in patients with FIGO stages I-II and III-IV

The 5-year OS rates for patients with an OTT ≤ 56 days compared with > 56 days with FIGO stages I-II and stage III-IVA were 70% and 65% (p = 0.002; Figure 1C) compared with 43% and 42% (p = 0.67; Figure 1D), respectively. The 5-year CSS rates for the ≤ 56 days compared with > 56 days OTT subgroups for patients with FIGO stages I-IIB and III-IVA were 82% and 73% (p < 0.001; Figure 1E) compared with 50% and 49% (p = 0.92; Figure 1F), respectively.

The subgroup analyses of CSS and OS for patients with FIGO stage I-IIB and III-IVA after adjustment for confounding factors, such as age, histology, use of brachytherapy, and use of chemotherapy, revealed a significant detrimental effect of prolonged OTT on OS (HR = 1.22, 95% CI = 1.03-1.46, p = 0.02; Table 4) and CSS (HR = 1.54, 95% CI = 1.22-1.93, p = 0.001; Table 4) only in patients with FIGO stage I-IIB disease. Prolonged OTT as a detrimental factor on survival was also noted in the subgroup of patients with FIGO stage IB (HR = 1.11, 95% CI = 1.01-1.62, p = 0.56), IIA (HR = 1.56, 95% CI = 1.03-2.37, p = 0.04), and IIB (HR = 1.28, 95% CI = 1.01-1.62, p = 0.04) disease (Supplementary Table 2).

**Table 4:** Impact of the duration of the entire radiation therapy course according to Cox’s proportional hazards model stratified by FIGO stage: prior to matching

	Overall survival				Cancer-specific survival				
	Adjusted HR	95% CI lower	95% CI upper	*p-*value^a^	Adjusted	HR	95% CI lower	95% CI upper	*p-*value^a^
**FIGO stage I**–**IIB**				0.02					0.001
≤ 56 days (Reference)	1.00				1.00				
> 56 days	1.22	1.03	1.46		1.54		1.22	1.93	
**FIGO stage III**–**IVA**				0.13					0.39
≤ 56 days (Reference)	1.00				1.00				
> 56 days	0.84	0.66	1.05		0.90		0.69	1.16	

FIGO: International Federation of Gynecology and Obstetrics; CI: confidence interval; HR: hazard ratio.

^a^Adjusted for age, FIGO stage, histology, the requirement for brachytherapy and chemotherapy.

### Impact of prolonged OTT on CSS and OS in patients with FIGO stage I-II after propensity score matching (PSM)

PSM according to age, histology, lymph node status, use of chemotherapy, and use of brachytherapy was performed for the patients with FIGO stage I-II disease (*n* = 1134) to generate a well-matched analysis of OS and CSS between patients with an OTT ≤ 56 days (*n* = 295) and those with an OTT > 56 days (*n* = 839). The result indicated that an OTT > 56 days remained a significant prognostic factor for poor CSS in the subgroup of patients with FIGO stage I-II disease (HR = 1.36, 95% CI = 1.05-1.75, p = 0.02; Table 5).

**Table 5:** Impact of the duration of the entire radiation therapy course on patients with FIGO stage I and II according to Cox’s proportional hazards model after propensity score matching

	Overall survival				Cancer-specific survival			
	Adjusted HR	95% CI lower	95% CI upper	*p-*value	Adjusted	95% CI lower	95% CI upper	*p*-value
**FIGO stage I**–**IIB**				0.23				0.02
≤ 56 days (Reference)	1.00				1.00			
> 56 days	1.13	0.93	1.38		1.36	1.05	1.75	

FIGO: International Federation of Gynecology and Obstetrics; CI: confidence interval; HR: hazard ratio.

## DISCUSSION

Prolonged OTT has been correlated with poorer pelvic control and CSS in cervical cancer patients receiving definitive RT alone in several retrospective studies [2-4, 13, 14]. According to Petereit et al., a prolonged OTT of > 55 days was an adverse factor for pelvic control and CSS in a retrospective cohort of 209 cervical cancer patients treated with external beam radiotherapy (EBRT) and low-dose rate brachytherapy [3]. Additionally, Chen et al. reported that a prolonged OTT, of > 63 days, was associated with poor pelvic control and 5-year cause-specific survival in their series of cervical cancer patients receiving EBRT and high-dose rate brachytherapy [2]. However, these studies predominantly predate the era of CCRT as the standard treatment for cervical carcinoma [12].

For cervical cancer patients treated with CCRT, Nugent et al. demonstrated correlations of poorer progression-free survival (PFS) and OS with a longer time to RT completion [15]. Another retrospective analysis of women treated with weekly cisplatin and pelvic RT according to the GOG 165 protocol found that treatment delay (> 8 weeks) was associated with worse PFS and OS [9, 12, 16]. Song et al. revealed that the 3-year pelvic failure-free rate for patients with an OTT > 56 versus ≤ 56 days was 26% versus 9% (p = 0.04), respectively, but that treatment time delay was not associated with the 3-year distant failure rate or the disease-specific mortality rate [12]. According to Shaverdian et al., the subgroup analysis performed in their study revealed that treatment delay did not predict in-field relapse, DFS, or OS in their CCRT cohort. The associations of prolonged treatment time in patients who received CCRT with PFS, DFS, or OS appear not to be consistent among previous studies [11, 12].

The aim of our study was to present results based on a nationwide cohort (Taiwan Cancer Registry Database) to reveal the prognostic value of the OTT on the 5-year CSS and OS of cervical cancer patients treated mainly with concurrent chemotherapy (71.3%) across all disease stages. A worse outcome with regard to crude CSS and OS, according to our analysis, was associated with a prolonged OTT. After adjusting for age, FIGO stage, histology, use of brachytherapy, and use of chemotherapy, prolonged OTT remained a significant prognostic factor for poor CSS. MVA revealed that prolonged OTT was a prognostic factor for both poor CSS and OS across the whole cohort, and the HRs for CSS and OS in patients treated with an OTT of > 56 days were 1.33 (p < 0.001) and 1.15 (p = 0.05), respectively. We further analyzed the impact of prolonged OTT on CSS and OSS in the patients with stage I–IIB and stage III–IVA using Cox’s proportional hazards model and a PSM method. Our results revealed that prolonged OTT remained a significant prognostic factor for poor CSS in patients with FIGO stage I-IIB disease, but not in patients with FIGO stage III-IVA disease.

According to Song et al., prolonged OTT increased the 3-year pelvic failure rate (OTT > 56 days vs. ≤ 56 days: 26% vs. 9%; p = 0.04), but the increase in pelvic recurrence could not be translated to the overall distant failure rate and disease-specific mortality probability due to the use of chemotherapy [12]. Their results also revealed that the 3-year distant failure rate and disease-specific mortality probability for patients with a total RT treatment time of > 56 days compared with ≤ 56 days was 28% and 29% compared with 26% and 29%, respectively. Our present study revealed that an OTT ≤ 56 days was correlated with better CSS, and the subgroup analysis indicated that patients with stage I-IIB disease benefit the most. In our study, the 3-year CSS for all FIGO stage patients with an OTT > 56 days and an OTT ≤ 56 days was 78% and 82% (p < 0.001), respectively. The 3-year CSS for FIGO stage I-IIB patients with an OTT > 56 days and an OTT ≤ 56 days were 79% and 87% (p < 0.001), respectively. The disparity between our study and that of Song et al. might be the result of a superior 3-year CSS in patients with a treatment time of < 56 days in our study. The present study included a large proportion of patients with stage I-IIB disease (71.6%); better pelvic control as a result of a shortened treatment time might also affect CSS in such patients.

Shaverdian et al. performed a crude analysis revealing that patients with an OTT > 56 days experienced greater in-field recurrence (HR = 2.170, p = 0.004), poorer DFS (HR = 1.737, p = 0.002), and poorer OS (HR = 1.804, p = 0.001) compared with patients with an OTT ≤ 56 days [11]. However, the adjusted analysis revealed no significant difference in in-field recurrence, DFS, or OS between patients with an OTT > 56 days and those with an OTT ≤ 56 days. The study included a smaller cohort, shorter median OTT (55 days in the RT alone group and 51 days in the CCRT group), and only 47 patients (28.3%) with an OTT > 56 days. The median OTT in our study was 59 days, and 993 patients (38.2%) with an OTT > 56 days were included. A larger cohort and more balanced distribution between patients with an OTT > 56 days and those with an OTT ≤ 56 days might have decreased the attribution of other prognostic factors on the adjusted analysis of CSS and OS in our study.

In the present study, there was a significant CSS disadvantage in FIGO stage I-IIB patients with an OTT > 56 days compared with an OTT ≤ 56 days on multivariate analysis with PSM (HR = 1.36; 95% CI = 1.05-1.75, p = 0.02) but was less of an OS advantage (p = 0.23; Table 5). In patients with advanced FIGO stage III-IVA, the OTT was not related to survival under chemoradiation. A similar trend was also observed when comparing an OTT of < 63 days versus ≥ 63 days among patients with FIGO stages IIB and III [2]. No significant association was found between more advanced stages and longer treatment duration. This subgroup analysis is in agreement with previous studies using radiation alone [3, 4]. In patients with stage III-IVA disease, the lack of a correlation between treatment time and survival was superseded by a positive correlation observed in the patients receiving chemotherapy and a higher radiation dose, as described by Fyles et al. [17].

The retrospective nature of our study potentially presents various confounders. Moreover, this study was based on the Taiwan Cancer Registry Database and might contain limited information regarding treatment, such as the hemoglobin level; the imaging modality, such as PET or MRI; the status of radiation boost; the regimens of chemotherapy; and individual reasons for RT interruption. Single-agent cisplatin-based chemoradiation revealed comparable efficacy in the treatment of cervical cancer and with less toxicity than two combination regimens, fluorouracil/cisplatin and topotecan/cisplatin [10], as recommended under the guidelines of the Taiwan Cooperative Oncology Group. The most common chemotherapy regimen for CCRT included cisplatin, fluorouracil, carboplatin, ifosfamide, and topotecan in this nationwide cohort according to the Taiwan National Insurance Research Dataset [18]. In a previous large series in Taiwan, Chen et al. analyzed the possible reasons for prolonged OTT, and they found the most common reason was increased interval between EBRT and HDRICB [2]. Severe hematological toxicity related to CCRT, machine breakdown, and personal reasons were also noted as causes of prolonged OTT [3]. The treatment gap between external beam radiation and intracavitary insertion of brachytherapy would be the most common potential reason of prolonged OTT in the RT era [2, 3, 13]. However, there was more grade 3+ acute toxicity mentioned in the concurrent chemoradiation era [12, 13]. About 10% of patients experienced grade 3 or worse acute toxicity in the longer OTT group, compared with 2% of grade 3 or more in the shorter OTT group [12]. Nevertheless, a large patient number, MVA, and subgroup comparison after PSM would decrease the attribution of those limitations. In conclusion, we compared the impact of the OTT on the 3- and 5-year CSS and OS across all FIGO stages in the nationwide population cohort during the CCRT era. Inferior CSS was associated with a prolonged OTT in the total cohort analysis. A further subgroup analysis revealed a significant improvement in the CSS by an OTT of ≤ 56 days only in patients with stage I-IIB disease. The completion of an RT course within 56 days is still warranted in the CCRT era, particularly in stage I-IIB patients.

## MATERIALS AND METHODS

### Study population

The national Taiwan Cancer Registry Database was screened for patients diagnosed with cervical cancer (including carcinoma *in situ*) between January 2007 and December 2013; in total, 27,395 evaluable patients were identified. The Taiwan Cancer Registry Database includes all newly diagnosed cancer cases in Taiwan. This study is part of the subgroup analysis for the Taiwan Core Measurement Indicators of Common Cancers project and was reviewed and approved by the Institutional Review Board of the Taiwan National Health Research Institutes.

### Cohort selection

The final cohort consisted only of patients who were treated with definitive curative chemoradiation/radiation. In total, 18,427 patients were excluded, because their pathology was recorded as carcinoma *in situ* or missing. Patients with initial surgery, distant metastatic disease, undetermined histology or histology other than squamous cell, adenocarcinoma, or neuroendocrine tumors, and those with an unknown or insufficient radiation dose, as defined by the Cancer Registry Database coding key, were also excluded. The definition of an insufficient radiation dose limited the cumulative dose to < 3,400 cGy and/or < 6,000 cGy without the addition of a brachytherapy boost. All patients included in the analysis had been labeled with a FIGO stage. American Joint Committee on Cancer staging was not mandatory in the Cancer Registry Database until late 2008. The remaining patients were stratified into two groups: those with an overall treatment tine (OTT) of ≤ 56 days and those with an OTT > 56 days. According to the coding principles of Taiwan Cancer Registry, the last day of the radiation treatment course was defined as the last day of external beam radiation (EBRT) in those patients receiving EBRT alone as the primary treatment and the last of the completion date of EBRT and brachytherapy in the subgroup receiving both EBRT and intracavitary brachytherapy as the primary treatment. OTT was calculated from the first day of EBRT to the last day of the radiation treatment course.

The primary endpoint of our analysis was cancer-specific survival (CSS). OS was measured from the date of diagnosis, as determined by the vital status obtained from the Taiwan Cancer Registry Database and the government database maintained by the Ministry of the Interior.

### Prognostic variables

Clinical variables included in the statistical analyses were patient age, tumor histology, FIGO stage, pelvic lymph node status, tumor size, requirement for a brachytherapy boost, requirement for chemotherapy, and OTT.

### Statistical analysis

Demographic data are presented in Table 1, along with the percentages of patients in each subgroup. The χ^2^ (Fisher’s exact) and independent *t*-tests were used to assess differences in demographic characteristics between patients with a treatment time of ≤ 56 days versus > 56 days. Potential prognostic variables were evaluated by univariate analysis (UVA) and multivariate analysis (MVA), using Cox’s proportional hazards models. Cox’s proportional hazards models were also used to examine the association between OTT and OS/CSS while controlling for other clinical (e.g., stage, histology) and demographic (e.g., age) variables. OS and CSS were estimated using the Kaplan–Meier method, with differences assessed using the log-rank test. All tests were two-tailed, and a *p*-value < 0.05 was considered to indicate statistical significance. All calculations were performed using SAS (ver. 9.3; SAS Institute Inc., Cary, NC, USA) and SPSS (ver. 21.0; SPSS Inc., Chicago, IL, USA) software packages.

The adjustment of observed effects in retrospective studies is a key part of data analysis, because the influences of potential confounders can bias effect estimates. To reduce the impact of subgroup selection bias, we adjusted the patient characteristics using propensity score matching (PSM). We derived the propensity score from a logistic regression model using variables associated with treatment time (age, FIGO stage, histology, lymph node status, chemotherapy, and brachytherapy) to achieve maximal group similarity for these parameters, rather than based on their statistical significance. After estimation of the propensity score, we matched patients (1:1) according to their propensity score ±0.05 using the nearest-neighbor matching method. Then, we examined the balance among all observed covariates and found that almost no imbalances remained, as assessed by statistical tests.

## SUPPLEMENTARY MATERIALS TABLES



## Abbreviations

CCRT: concurrent chemoradiotherapy
CSS: cancer-specific survival
DFS: disease-free survival
EBRT: external beam radiotherapy
FIGO: International Federation of Gynecology and Obstetrics
HR: hazard ratio
OS: overall survival
OTT: overall treatment time
PFS: progression-free survival
PSM: propensity score matching
RT: radiotherapy
