# Supplementary material for: The prognostic impact of overall treatment time on disease outcome in uterine cervical cancer patients treated primarily with concomitant chemoradiotherapy: a nationwide Taiwanese cohort study
Source: Oncotarget. 2017 Jul 27;8(49):85203–13. doi: 10.18632/oncotarget.19617 (PMC5689603; doi:10.18632/oncotarget.19617)
Supplement: Supplementary file 1 [file oncotarget-08-85203-s001.pdf]

The prognostic impact of overall treatment time on disease outcome in uterine cervical cancer patients treated primarily with concomitant chemoradiotherapy: a nationwide Taiwanese cohort study

SUPPLEMENTARY MATERIALS

Supplementary Table 1: Multivariate analyses of the overall treatment time and clinical outcome in the subgroup of the patients with definitive concurrent chemoRT and brachytherapy (Cox regression *p* values and associated HR)

Variable	Adjusted HR	95% CI lower	95% CI upper	P value[a]
A. Overall survival				
≤ 56 days (Reference)	1			
> 56 days	1.29	1.05	1.58	0.01
B. Cancer-specific survival				
≤ 56 days (Reference)	1			
> 56 days	1.29	1.02	1.62	0.03

RT: radiotherapy, CCRT: curative concurrent chemoradiotherapy, HR: hazard ratio, CI: confidence interval.
[a]Adjusted for age, FIGO stage, histology, requirement for brachytherapy, and requirement for chemotherapy.

Supplementary Table 2: Subgroup analysis of impact of the duration of the entire radiation therapy course according to Cox's proportional hazards model, stratified by FIGO stage

Variable	Adjusted HR	95% CI lower	95% CI upper	P value[a]
A. Stage IA				
≤ 56 days (Reference)	1			
> 56 days	0.78	0.08	8.07	0.83
B. Stage IB				
≤ 56 days (Reference)	1			
> 56 days	1.11	0.78	1.57	0.56
C. Stage IIA				
≤ 56 days (Reference)	1			
> 56 days	1.56	1.03	2.37	0.04
D. Stage IIB				
≤ 56 days (Reference)	1			
> 56 days	1.28	1.01	1.62	0.04

FIGO: International Federation of Gynecology and Obstetrics; CI: confidence interval; HR: hazard ratio.
[a]Adjusted for age, FIGO stage, histology, the requirement for brachytherapy and chemotherapy.
